# Longitudinal Changes of Regional Cerebral Blood Flow on a Single-Photon Emission Computed Tomography (SPECT) Scan in a Patient With Schizophrenia Having Cotard’s Syndrome

**DOI:** 10.7759/cureus.58263

**Published:** 2024-04-14

**Authors:** Konoka Nomura, Ryota Kobayashi, Toshinori Shirata, Keisuke Noto, Akihito Suzuki

**Affiliations:** 1 Psychiatry, Yamagata University Faculty of Medicine, Yamagata, JPN

**Keywords:** longitudinal change, schizophrenia, rcbf, spect, cotard’s syndrome

## Abstract

Cotard’s syndrome is a rare clinical condition characterized by the presence of nihilistic delusions, delusions of immortality, depressive mood, and anxiety. Longitudinal changes in regional cerebral blood flow (rCBF) obtained under different conditions with and without Cotard’s syndrome have rarely been reported in the literature. We report a case of a patient with Cotard’s syndrome in whom longitudinal rCBF was assessed using single-photon emission computed tomography (SPECT).

The patient was a 52-year-old man suffering from schizophrenia and mild mental retardation. He was transported to our hospital because of lumbar fractures caused by a suicidal attempt. In the second week after admission, he displayed Cotard’s syndrome, i.e., nihilistic delusions, suicidal thoughts, and depressive mood. SPECT with 99mTc-ethyl cysteinate dimer was performed, and the rCBF increased in the bilateral prefrontal cortex but decreased in the occipital and parietal lobes. He was treated with pharmacotherapy mainly using lurasidone, and his Cotard’s symptoms disappeared. SPECT was performed again. The increased rCBF in the bilateral prefrontal cortex and the decreased rCBF in the right occipital and parietal lobes were improved.

The present case suggests that increased rCBF in the prefrontal cortex and decreased rCBF in the right occipital and parietal lobes are associated with the development of Cotard’s syndrome.

## Introduction

Cotard’s syndrome is a rare clinical condition that is characterized by the presence of nihilistic delusions, delusions of immortality, suicidal thoughts, analgesia, depressive mood, and melancholic anxiety [1]. This syndrome is observed mainly in patients with severe depression but has been reported in patients with various organic brain illnesses and psychiatric disorders such as schizophrenia [1]. Neuroimaging studies have been performed to investigate the pathophysiology of Cotard’s syndrome [1,2]. Longitudinal changes in regional cerebral blood flow (rCBF) obtained under different conditions with and without Cotard’s syndrome have rarely been reported in the literature [3-7]. We report a case of a patient with Cotard’s syndrome in whom longitudinal rCBF was assessed using single-photon emission computed tomography (SPECT).

## Case presentation

The patient was a 52-year-old, right-handed man with schizophrenia and mild mental retardation. He had developed schizophrenia in his 30s. After several psychotic episodes, he had been in a residual state without hallucinations and delusions under treatment with aripiprazole (24 mg/day), risperidone (3 mg/day), and levomepromazine (130 mg/day) at a psychiatric hospital. Flunitrazepam (2 mg/day) and suvorexant (20 mg/day) were used for the management of his insomnia. He also received metformin (750 mg/day), teneligliptin (20 mg/day), and canagliflozin (100 mg/day) for the treatment of diabetes mellitus. His family history revealed that his mother was suffering from schizophrenia.

**Figure 1:**
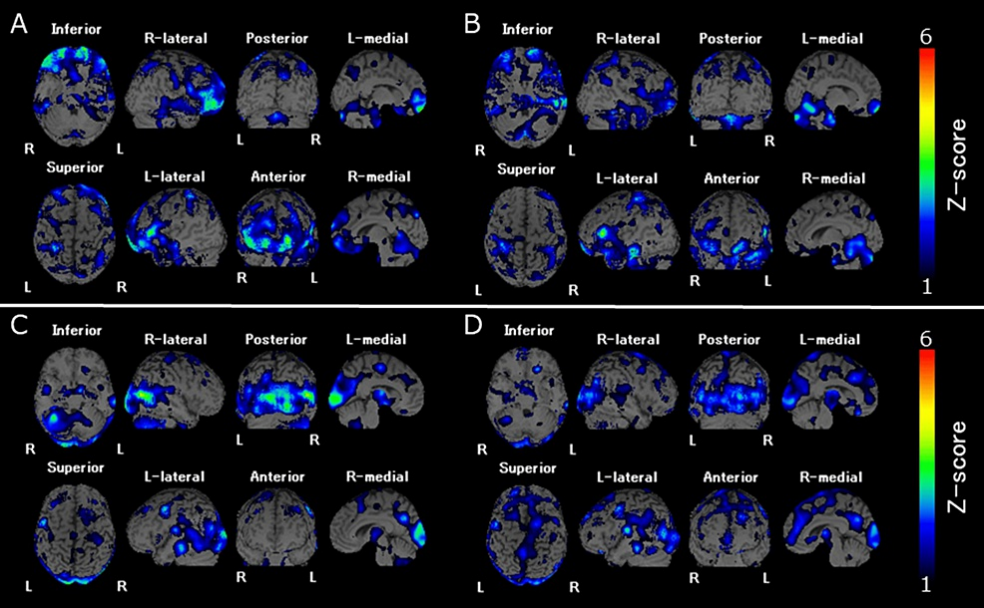
Increase in rCBF with (A) and without Cotard’s syndrome (B), and decrease in rCBF with (C) and without Cotard’s syndrome (D). rCBF: regional cerebral blood flow; SPECT: single-photon emission computed tomography.

The patient was transported to our hospital because of lumbar fractures caused by a suicide attempt. He was admitted to the Department of Psychiatry after undergoing surgical posterior spinal fusion and short-term pain relief in the Department of Orthopedics. On admission, he had auditory hallucinations of his mother’s voice, persecutory delusions, blunted affect, and motor retardation. He mentioned that he was depressed and regretted shoplifting a few years ago; thus, he had no reason to live. The same pharmacological therapy as prescribed by the previous hospital was continued for the treatment of schizophrenia. In the second week after admission, he mentioned that he was dead, his heart had stopped, his limbs were cold, and his body was melting with swoosh sounds. The results of laboratory blood tests and brain magnetic resonance imaging were unremarkable. In the third week, SPECT with 99mTc-ethyl cysteinate dimer was performed in order to differentiate between cerebrovascular diseases and neurodegenerative diseases. SPECT image acquisition and reconstruction were performed the same as in a previous study [8]. The rCBF increased in the bilateral prefrontal cortex (Figure 1A) but decreased in the occipital and parietal lobes, predominantly on the right side (Figure 1C). He was treated with lurasidone (80 mg/day), flunitrazepam (2 mg/day), and lemborexant (5 mg/day). His nihilistic delusions, auditory hallucinations, suicidal thoughts, and depressive mood gradually disappeared by the sixth week, but blunted affect and avolition remained. In the ninth week, SPECT was performed again; the increased rCBF in the bilateral prefrontal cortex (Figure 1B) and the decreased rCBF in the right occipital and parietal lobes (Figure 1D) were improved, although they were not completely normalized. In the 15th week after admission, he was transferred to the psychiatric hospital that he had been admitted to before.

## Discussion

The present case showed increased rCBF in the bilateral prefrontal cortex in the presence of Cotard’s syndrome (i.e., nihilistic delusions, suicidal thoughts, and depressive mood), which improved after the disappearance of the syndrome. Five reports have described longitudinal changes in rCBF in patients with Cotard’s syndrome (Table 1) [3-7], and the most consistent findings in these cases, together with the present case, are alterations in rCBF in the prefrontal cortex. Restrepo-Martínez et al. suggested that Cotard’s syndrome is developed by two factors: anomalous perception and abnormal reasoning; dysfunction of the prefrontal cortex creates a foundation for abnormal reasoning [2]. Previous reports suggested that rCBF in the frontal cortex was decreased in patients with Cotard's syndrome [3,5,7], whereas in the present case, this was observed to increase. The mechanism underlying this discrepancy remains unclear, but it is conceivable that abnormalities of rCBF in the frontal cortex are implicated in Cotard's syndrome. Meanwhile, in this case, decreased rCBF in the right occipital and parietal lobes was observed in Cotard’s syndrome. Previous reports have suggested that the right hemisphere plays an important role in feelings of familiarity with the self and ownership of the body and proposed that dysfunction of the right hemisphere is related to Cotard’s syndrome [9]. Taken together, increased rCBF in the prefrontal cortex and decreased rCBF in the right occipital and parietal lobes might be associated with the development of Cotard’s syndrome, as observed in this case.

**Table 1:** Studies examining longitudinal changes in rCBF in patients with Cotard’s syndrome ECT: electro-convulsive therapy; rCBF: regional cerebral blood flow.

Study	Age (yo), sex	Background diseases	Treatments	Findings of rCBF with and without Cotard's syndrome
Petracca et al. (1995) [7]	56, male	Major depression	ECT	Decreased rCBF in the bilateral dorsolateral frontal lobe, fronto-parietal medial cortex, basal ganglia, and thalamus during Cotard's syndrome. Improvement of rCBF in these areas after the syndrome.
Hashioka et al. (2002) [5]	57, female	Major depression	Pharmacotherapy (sulpiride, imipramine)	Decreased rCBF in the bilateral frontal lobes during Cotard's syndrome. Improvement of rCBF in the frontal lobes after the syndrome.
Caliyurt et al. (2004) [3]	27, male	Schizophreniform disorder	ECT	Decreased rCBF in the left temporal, inferior frontal, and parietal lobes during Cotard's syndrome. Improvement of rCBF in the inferior frontal and parietal lobes, and residual decreased rCBF in the left temporal lobe after the syndrome after the syndrome.
De Risio et al. (2004) [4]	43, male	Major depression with psychotic features	Pharmacotherapy (clozapine, fluvoxamine, imipramine)	No rCBF abnormalities.
Maruo et al. (2016) [6]	62, female	Treatment-resistant psychotic major depression	Pharmacotherapy (mirtazapine, lorazepam, quetiapine, olanzapine, aripiprazole, duloxetine, pramipexole)	Decreased rCBF in bilateral parieto-occipital cortices during Cotard's syndrome. Similar results after the syndrome.

SPECT studies for Cotard’s syndrome have produced discrepant results regarding the locations of rCBF, i.e., the temporal lobe, parietal lobe, occipital lobe, basal ganglia, thalamus, or insular cortex [2-7]. These discrepancies indicate that the mechanism of Cotard’s syndrome is highly complicated and that other factors including age, sex, chronic/acute cases, organic comorbidities, and treatments such as electroconvulsive therapy and pharmacotherapy influence rCBF in Cotard’s syndrome (Table 1) [10,11]. In the present case as well, the possibility that lurasidone influenced directly rCBF in several brain regions of the patient, irrespective of the improvement of Cotard syndrome, cannot be excluded, although there have been no studies examining the direct effects of lurasidone on rCBF in schizophrenic patients. Alternatively, subtypes of Cotard’s syndrome (i.e., psychotic depression, the pure forms of nihilistic delusions without affective symptoms, and a mixed group of symptoms of anxiety, depression, and auditory hallucinations), as suggested by Berrios and Luque [12], may be implicated in the discrepancies. Thus, larger and controlled study designs such as cross-sectional studies and longitudinal studies are warranted to elucidate the rCBF in patients with Cotard’s syndrome.

## Conclusions

We reported the case of a patient with Cotard’s syndrome in whom longitudinal rCBF was assessed using SPECT. After treatment with pharmacotherapy, the patient's Cotard’s syndrome improved, correlating with changes in rCBF in several brain areas. The present case suggests that increased rCBF in the prefrontal cortex and decreased rCBF in the right occipital and parietal lobes are associated with the development of Cotard’s syndrome.
